# NEWS

**DOI:** 10.7189/jogh.02.020202

**Published:** 2012-12

## THE BILL AND MELINDA GATES FOUNDATION

• The company Cepheid said that it would be working with the Bill & Melinda Gates Foundation, PEPFAR, USAID and UNITAID to make its diagnostic kit for tuberculosis available at reduced prices in low and middle income settings where the burden of the disease is large. The representatives of Cepheid said that the four organizations committed to funding the purchase of the Xpert MTB/RIF test in 145 countries with largest burden of tuberculosis. (*Reuters*, 7 Aug 2012)

• The Bill & Melinda Gates Foundation has launched Round 10 of its Grand Challenges Explorations, a funding initiative worth US$ 100 million to encourage innovative research in global health and development. Applications are usually only two pages, with the initial grant budget of US$ 100 000 to demonstrate the potential. Applicants from low– and middle–income countries are particularly encouraged to apply. (*BMGF*, 5 Sep 2012)

• Following The Giving Pledge call from Mr Bill Gates and Mr Warren Buffet to other billionaires to consider donating half of their wealth to charity, further eleven billionaires added their names to the list – bringing the current total to 92. The new members of this group include Reed Hastings, the CEO of Netflix; Gordon Moore, co–founder of Intel; and Charles Bronfman, the head of Seagram Co. (*AFP*, 19 Sep 2012)

• Mr Bill Gates has signed an agreement with the Islamic Development Bank, situated in Jeddah. The agreement is believed to be worth more than US$ 250 million and it should serve the aim to gain Middle East support in the fight to eradicate polio. According to Mr Ahmad Mohamed Ali, who is the chairman and the president of Islamic Development Bank, this five–year programme should cover projects mainly in Pakistan, Afghanistan and Nigeria. (*Arabian Business*, 8 Oct 2012)

• The Bill and Melinda Gates Foundation joined forces with Mr Aliko Dangote's foundation to fight polio, as the disease resurged in Nigeria. Mr Dangote, a Nigerian businessman considered to be Africa's richest man by the Forbes magazine, announced this alliance in Kano, which is Nigeria's second largest city. Only three countries are considered to still be vulnerable to endemic polio – Nigeria, Pakistan and Afghanistan. The four–year alliance should provide funding, equipment and technical support to strengthen polio immunisation in Kano region. (*AFP*, 27 Nov 2012)

## THE GAVI ALLIANCE

• Yemen has announced plans to vaccinate its one million–strong birth cohort against the diarrhoea caused by rotavirus. The campaign will be supported by the Global Alliance for Vaccines and Immunisation (GAVI). Yemen's child mortality is still as high as 77 deaths for every 1000 live births, while more than 46% of the population live with less than US$ 2 a day. (*The Guardian*, 1 Aug 2012)

• Scientists claim to have developed the first vaccine for hepatitis C, thought to affect up to 200 million people worldwide. Hepatitis C is an infectious disease that most often targets the liver and it is caused by the hepatitis C virus (HCV). According to researchers from the Burnet Institute in Melbourne, a preventative vaccine would have the potential to have a significant global health impact. (*Business Standard*, 16 Aug 2012)

• In September, European Union health officials suggested that all girls in Europe should be immunized against the human papillomavirus (HPV) that causes cervical cancer, and that the current vaccine coverage rates are far too low. The European Centre for Disease Prevention and Control (ECDC) said that only 19 out of 29 countries in the region had introduced HPV vaccine, and that vaccination rates were as low as 17% in some of those who started vaccine roll–out. (*Reuters*, 4 Sep 2012)

• Following the World Health Organization's recommendation to include rotavirus vaccination in their national immunization programme, 41 countries have already introduced rotavirus vaccines. However, Asian countries are still lagging behind and only two countries in Asia – Philippines and Thailand – are currently following this advice. WHO suggested that the price continues to be an important barrier to global scale–up. (*IRIN*, 7 Sep 2012)

Dengue fever threatens nearly half of the world's population. The leading candidate vaccine against the disease has shown to be only 30% effective in its first large clinical trial, although clearly representing an important milestone in the 70–year quest to develop such a vaccine. The study’s goal of 70% effectiveness hasn't been reached, nor has the statistical significance between the two arms of the trial, but the study still demonstrated that a safe and effective inoculation against dengue is feasible. (*New York Times*, 10 Sep 2012)

## THE WORLD BANK

• “The Washington Consensus” has set the fundamental requirements for the World Bank's developmental aid since the late 1980s. It has controversially insisted on deregulation, privatisation and an overall reduced role of the state in industry and domestic market forces. These policies were designed to encourage development of neo–liberal economies suitable for integration into international markets, but have also attracted criticism for driving numerous diverse, multi–cultural societies into a single economic model. Under new leadership from the outspoken Korean–American Jim Yong Kim, the bank aims to adopt evidence–based policy on “real world analysis”, and to disband the historical myth that reducing big–government control automatically improves the standard of living and ignites development. Kim is renowned for challenging conservative economic ideas and it is hoped that he could strengthen the role of the World Bank in global development. (*The Guardian*, 14 Jun 2012)

• For the first time, a physician and development expert has become the President of the World Bank: Jim Yong Kim left his post of the President of Dartmouth College to continue his lifelong work in global poverty reduction and economic development. Mr Kim has wide experience in health care delivery, from co–founding the organisation Partners in Health to spearheading ambitious campaigns at the WHO. He faces significant challenges in his new role, having to balance competing demand for resources to target economic growth with the need to improve health and education programmes. During his future at the World Bank, Kim’s vision is to “get better, more focused and effective, and deliver on the Bank's promise: our dream is a world free of poverty.” (*The Lancet*, 2 Jul 2012)

• The new World Development Report from the World Bank suggests that some 600 million new jobs may be needed worldwide in the next 15 years to absorb a growing workforce. The need will be the greatest in Asia and Sub–Saharan Africa. The experts recommended a three–stage approach by governments, urging them to develop and implement policy fundamentals that include macroeconomic stability, a business–friendly environment, investments in human capital and the rule of law. In the second step, they should design labour policies to ensure that growth translates into employment opportunities. Finally, they should identify the jobs that support development, removing the obstacles from creating those jobs in the private sector. (*The Guardian*, 2 Oct 2012)

• Health campaigners from around the world have been trying to influence the World Bank’s president, Jim Yong Kim, urging him to support universal health care coverage in low– and middle–income countries. More than 100 organisations signed an open letter, stating that now is the moment “to play a truly progressive and transformative role in health, by supporting countries to achieve universal health coverage”. (*BMJ*, 12 Oct 2012)

• Details of World Bank–financed contracts will become available online, which is as part of a global initiative to fight corruption. The move should also encourage governments to disclose their deals with private companies. The World Bank Institute, which is the capacity development branch of the World Bank, said the organisation would ensure that its own contracts are disclosed while also helping countries open their books. The move should strengthen the nascent Open Contracting Initiative, which is a global campaign to increase disclosure and participation in public contracting. Governments around the world are estimated to spend around US$ 10 trillion each year contracting private companies, but their deals are rarely transparent although in many poor countries procurement accounts for two thirds of all public spending. (*The Guardian*, 26 Oct 2012)

## UNITED NATIONS

• More than 330 000 children are still being born with HIV every year. The UN set a goal to eliminate these infections in babies by 2015, but it appears to be very far from reaching this target. The problem partially lies in targeting mother–to–child transmission only, leaving many HIV–positive women without treatment for themselves. This results in a high mortality rate among these women, leaving many newborns orphaned within two years. Malawi leads the way to address this problem with plan B+, a programme that puts HIV–positive pregnant women onto lifelong treatment. Botswana, Rwanda and many others are expected to follow, although these programmes would come with a massive global price tag that would need to be covered. (*The Guardian*, 25 Jul 2012)

• UN Secretary–General, Mr Ban Ki–moon, has appointed a high–level panel to develop the post–Millennium Development Goals agenda. The panel will be meeting at the sidelines of the U.N. General Assembly. Mr Jeffrey Sachs, who is a special adviser to Mr Ban on the Millennium Development Goals, suggested creating four big pillars: ending extreme poverty, social inclusion, environmental sustainability and good governance. (*Devex*, 17 Sept 2012)

• The UN has warned that the world's grain reserves are dangerously low and severe weather in the United States or other food–exporting countries could trigger a major hunger crisis next year. This year the world will consume more food than it produces, largely because of extreme weather in the US and other major food–exporting countries. Oxfam announced last week that the price of key staples, which include wheat and rice, may double in the next 20 years. This would have very concerning consequences for people in low–resource settings who spend a large proportion of their income on food. (*The Guardian*, 13 Oct 2012)

• Two United Nations agencies launched a new initiative in October, called ‘m–Health’. They proposed to use mobile technology – particularly text messaging and applications – to help tackle non–communicable diseases. The ITU and the WHO will be expected to provide evidence–based and operational guidance to encourage future users, including national governments, to implement m–Health interventions to address prevention and treatment of non–communicable diseases and their common risk factors. This new initiative is initially expected to run for a four–year period and focus on both prevention and treatment of non–communicable diseases. (*UN News*, 17 Oct 2012)

• In May this year, Mr Ban Ki–moon, the UN Secretary General, named UK prime minister David Cameron, Liberian president Ellen Johnson Sirleaf, and President Susilo Bambang Yudhoyono, of Indonesia, as co–chairs of a high–level panel to advise him on the global development agenda after 2015. Further 26 members were then appointed on this panel to work on a report, setting out a “bold yet practical vision”. The lead author of this report will be Mr Homi Kharas, a former World Bank economist and presently working at the Brookings Institution in Washington, DC. (*The Guardian*, 31 Oct 2012)

## UN AIDS

• The Programme Coordinating Board of the Joint United Nations Programme on HIV/AIDS (UNAIDS) met for its 30th session in June 2012 to review progress and propose recommendations for the global fight against AIDS. While the Director Michel Sidibe estimated that mother–to–child transmission of HIV/AIDS would be eliminated by 2015, the Board recognised that more efforts were needed in other areas. The Board created a new partnership with UN Women in a bid to improve the HIV/AIDS response towards women. Moreover, it urged member states to adapt their national HIV/AIDS response programmes to the specific needs of the female population. It also called for the implementation of laws enabling the successful delivery of HIV prevention and treatment programmes. (*UNAIDS*, 8 Jun 2012)

• A mysterious new disease has left scores of people in Asia and several further cases in the United States with AIDS–like symptoms, even though they are not infected with HIV. Acquired immune deficiency in affected patients has not been inherited and occurs in adults, but doesn't spread through a virus, according to Dr Sarah Browne, a scientist at the National Institute of Allergy and Infectious Diseases. She believes that further cases may exist, but that they are likely mistaken for tuberculosis in some countries. (*Associated Press*, 22 Aug 2012)

• According to the joint report from the UNAIDS, Funders Concerned About AIDS (FCAA) and the European HIV/AIDS Funders Group (EFG), private AIDS–related funding from United States and European philanthropic donors totalled US$ 644 million in 2011. This is a 5% increase from 2010 and it was driven by donations from the Bill & Melinda Gates Foundation. Analysis also revealed that very few new funders seem to be entering the field of AIDS philanthropy. (*UNAIDS*, 8 Nov 2012)

• The US government spends about US$ 6.4 billion a year on preventing and treating HIV/AIDS in the developing world. It is thought that between 4 and 5 million AIDS patients depend mostly on US–originated aid for the AIDS medicines to keep them alive. Ambassador Eric Goosby, the acting head of the President’s Emergency Program on AIDS Relief (PEPFAR), has a unique opportunity to make that money do even more good by releasing the data that PEPFAR and its contractors have already collected. Such move would allow proper evaluation of this program and it “...could improve the efficiency, the quality and the accountability of the US’s most frequently praised foreign assistance program”. (*Center for Global Development*, 13 Nov 2012)

• In its 2012 report, UNAIDS praised India for doing “particularly well” in halving the number of newly infected HIV–positive adults between 2000 and 2009. India is presently a home to about 2.4 million people living with HIV, one million of whom are on anti–retroviral treatment. However, the Human Immunodeficiency Virus Type I (HIV–1) has been undergoing a process of fairly rapid viral evolution. The subtype C is responsible for nearly 99% of infections in India, but the scientists working at the Jawaharlal Nehru Centre for Advanced Scientific Research (JNCASR) in Bangalore have found a family of five new strains of HIV–1 subtype C. As two of those new strains seem to be outstripping the standard viral strain, this finding is of a special concern for Indian authorities and it may pose a larger challenge in the future. (*IPS*, 29 Nov 2012)

## UNICEF

• A new report from UNICEF, Pneumonia and Diarrhoea: Tackling the Deadly Diseases for the World’s Poorest Children, has again highlighted the potential to narrow the survival gap through increased commitment and funding. These two diseases account for nearly one–third of deaths among children under five globally, with 90% of these deaths in Sub–Saharan Africa and South Asia. The report, released shortly before the meeting of several world leaders to launch a new initiative for child survival, suggests that simple interventions have the potential to save millions of lives. These include exclusive breast–feeding of babies, access to soap, routine immunisation programmes, and the appropriate use of antibiotics and oral rehydration therapy. (*GAVI Alliance*, 8 Jun 2012)

• After experiencing a re–emergence of the polio virus seven years ago, Angola has now been polio–free for a full year. United Nations agencies reported that this success is the result of the improvement in the quality of polio campaigns with each round of immunization, moving the world closer to the ultimate target of global eradication of polio. (*UN News*, 10 Aug 2012)

• According to Dr Mickey Chopra, chief health officer at UNICEF, “an intense focus on countries with the highest levels of child mortality, combined with the availability of cheaper vaccines and medicines, can lead to a development breakthrough”. Dr Chopra said that appropriate investments at this point in time could lead to massive gains in reducing maternal and child mortality in the coming years. (*The Guardian*, 28 Aug 2012)

• GSK's experimental malaria vaccine showed an effectiveness of only 30% among African babies in a crucial clinical trial. The surprisingly poor result left uncertainties over the vaccine's future role in fighting the mosquito–borne disease. (*Reuters*, 9 Nov 2012)

• This year, an estimate of the burden of tuberculosis in children was included in the Global Tuberculosis Report for the first time. Although this first estimate was very uncertain and likely too low, its inclusion filled an important gap in global paediatric information on tuberculosis. The growing awareness of the burden of this problem among children was reflected at the International Union of Tuberculosis and Lung Health Conference held in Kuala Lumpur in November. (*MSF*, 26 Nov 2012)

## WORLD HEALTH ORGANIZATION

• Several recent articles by senior UK doctors questioned the legitimacy of some post–licensing drug trials, suggesting they were more of a marketing ploy than a scientific exercise. These studies are usually intended to improve understanding of the effects in the ‘real world’, away from the close monitoring and strict regimens of all previous drug trial phases. Typically, a new therapy is offered to patients as potentially superior to the gold standard treatment, although considerably more expensive. On completing the trial period, funding from the pharmaceutical company can be withdrawn, leaving either a health care system or the patient to cover the cost of new treatment. Worryingly, these tactics are often employed in low– and middle–income countries, where people have to pay for their own health care and where a desire to stay on the new therapy could cause “catastrophic health expenditure” for households. (*The Guardian*, 12 Jun 2012)

• Advances in medical technology have often bypassed the African continent, being too expensive for its nations. However, a new vaccine against meningitis, MenAfriVac, conjointly developed by the World Health Organisation (WHO) and PATH and priced at less than 50 cents per dose, may become affordable to most African countries. Bill and Melinda Gates Foundation donated US$ 50 million to support the development of this vaccine. It is largely aimed to eradicate the meningitis epidemics in Africa and the coverage recently reached the 100 millionth person, only two years after it was first administered. (*WHO*, 13 Jun 2012)

• A recent study published in the Lancet Infectious Diseases journal suggested that the estimated mortality from the H1N1 influenza pandemic in 2009 was 15 times greater than previously thought, increasing the WHO's estimate of 18 500 to 579 000. The WHO's estimate was derived solely from laboratory confirmed cases of H1N1 and was known to likely underestimate the true impact of the outbreak. H1N1 virus is a novel combination of human, swine and avian flu and uncertainties about its virulence and pathogenicity were a significant obstacle to the international effort to combat its spread. (*Reuters*, 26 Jun 2012)

• There is a growing concern that parasites may become more virulent because of climate change. This is a conclusion of a recent study showing that frogs suffer more fungal infections when exposed to unexpected swings in temperatures. It is hypothesized that parasites may be better at adapting to climatic shifts than the animals they live on, while cold–blooded creatures may also be more susceptible to parasites as temperature shifts than warm–blooded ones. How other parasites, such as malaria, might be affected by temperature swings that affect both its hosts, mosquitoes and humans, remains to be explored. (*Reuters*, 12 Aug 2012)

• The pharmaceutical giant Pfizer announced that the World Health Organization (WHO) has granted an expansion to the prequalification of its pneumococcal conjugate vaccine, Prevenar 13, to include adults 50 years of age and older. The 13–valent vaccine covering 13 different pneumococcal serotypes should demonstrate high effectiveness in protecting the elderly against pneumonia and invasive disease. (*PharmPro*, 11 Sep 2012)

